# Evaluation of deep learning estimation of whole heart anatomy from automated cardiovascular magnetic resonance short- and long-axis analyses in UK Biobank

**DOI:** 10.1093/ehjci/jeae123

**Published:** 2024-05-09

**Authors:** Marica Muffoletto, Hao Xu, Richard Burns, Avan Suinesiaputra, Anastasia Nasopoulou, Karl P Kunze, Radhouene Neji, Steffen E Petersen, Steven A Niederer, Daniel Rueckert, Alistair A Young

**Affiliations:** School of Biomedical Engineering and Imaging Sciences, King’s College London, 1 Lambeth Palace Rd, London SE1 7EU, UK; School of Biomedical Engineering and Imaging Sciences, King’s College London, 1 Lambeth Palace Rd, London SE1 7EU, UK; College of Mathematical Medicine, Zhejiang Normal University, Zhejiang, China; Cardiovascular Research Group, Puyang Institute of Big Data and Artificial Intelligence, Henan, China; School of Biomedical Engineering and Imaging Sciences, King’s College London, 1 Lambeth Palace Rd, London SE1 7EU, UK; School of Biomedical Engineering and Imaging Sciences, King’s College London, 1 Lambeth Palace Rd, London SE1 7EU, UK; School of Biomedical Engineering and Imaging Sciences, King’s College London, 1 Lambeth Palace Rd, London SE1 7EU, UK; School of Biomedical Engineering and Imaging Sciences, King’s College London, 1 Lambeth Palace Rd, London SE1 7EU, UK; MR Research Collaborations, Siemens Healthcare Limited, Camberley, UK; School of Biomedical Engineering and Imaging Sciences, King’s College London, 1 Lambeth Palace Rd, London SE1 7EU, UK; William Harvey Research Institute, NIHR Barts Biomedical Research Centre, Queen Mary University London, Charterhouse Square, London EC1M 6BQ, UK; Barts Heart Centre, St Bartholomew’s Hospital, Barts Health NHS Trust, West Smithfield, London EC1A 7BE, UK; Cardiac Electro Mechanics Research Group, National Heart & Lung Institute, Imperial College London, London W12 0NN, UK; Digital Twin Turing Research and Innovation Cluster, The Alan Turing Institute, London NW1 2DB, UK; Department of Computing, Biomedical Image Analysis Group, Imperial College London, London, UK; Institute for Artificial Intelligence and Informatics in Medicine, Klinikum Rechts der Isar, Technical University of Munich, Munich, Germany; School of Biomedical Engineering and Imaging Sciences, King’s College London, 1 Lambeth Palace Rd, London SE1 7EU, UK

**Keywords:** deep learning, dense segmentations, volume estimates, regression, risk factors, disease factors

## Abstract

**Aims:**

Standard methods of heart chamber volume estimation in cardiovascular magnetic resonance (CMR) typically utilize simple geometric formulae based on a limited number of slices. We aimed to evaluate whether an automated deep learning neural network prediction of 3D anatomy of all four chambers would show stronger associations with cardiovascular risk factors and disease than standard volume estimation methods in the UK Biobank.

**Methods and results:**

A deep learning network was adapted to predict 3D segmentations of left and right ventricles (LV, RV) and atria (LA, RA) at ∼1 mm isotropic resolution from CMR short- and long-axis 2D segmentations obtained from a fully automated machine learning pipeline in 4723 individuals with cardiovascular disease (CVD) and 5733 without in the UK Biobank. Relationships between volumes at end-diastole (ED) and end-systole (ES) and risk/disease factors were quantified using univariate, multivariate, and logistic regression analyses. Strength of association between deep learning volumes and standard volumes was compared using the area under the receiving operator characteristic curve (AUC). Univariate and multivariate associations between deep learning volumes and most risk and disease factors were stronger than for standard volumes (higher *R*^2^ and more significant *P-*values), particularly for sex, age, and body mass index. AUCs for all logistic regressions were higher for deep learning volumes than standard volumes (*P* < 0.001 for all four chambers at ED and ES).

**Conclusion:**

Neural network reconstructions of whole heart volumes had significantly stronger associations with CVD and risk factors than standard volume estimation methods in an automatic processing pipeline.

## Graphical Abstract

**Graphical Abstract:**
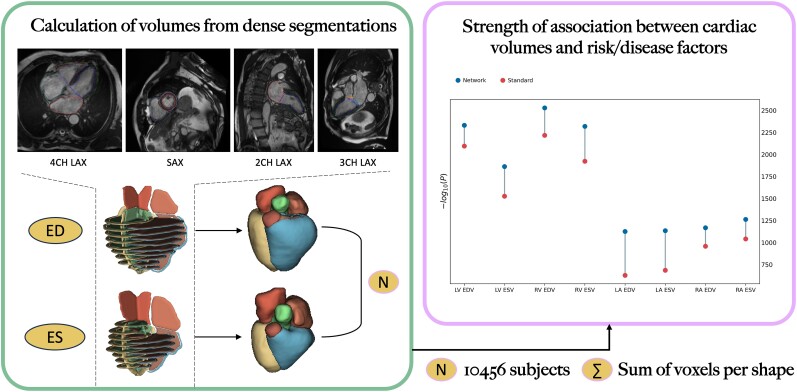

## Introduction

Accurate estimations of chamber volume for the right (RV) and left ventricles (LV) and atria of the heart are necessary for evaluation of cardiovascular disease (CVD). Cardiovascular magnetic resonance (CMR) has been shown to achieve good performance in heart volume estimation.^1^ However, standard measurements include only short-axis slices for estimation of right and left end-diastolic (ED) and end-systolic (ES) ventricular volumes, by multiplication of slice areas with the inter slice distance, whereas right and left atrial volumes are estimated from two-chamber and four-chamber long-axis views using single plane and biplane ellipsoidal approximation formulae.^2,3^ A more robust and accurate method for estimating atrial and ventricular volume, which utilizes all the short- and long-axis slices from typical CMR acquisitions, may enable better characterization of relationships between disease and anatomy.

Deep learning methods have recently enabled the reconstruction of 3D geometry and volume from sparse slices.^4–7^ A 3D U-Net was shown to accurately reconstruct left atrial shape and volume from two-chamber and four-chamber views^8^ and was recently extended to reconstruct whole heart anatomy from short- and long-axis views.^9^

The aim of this study was to adapt and evaluate this method in a large cohort study (UK Biobank) using a fully automated pipeline. We hypothesized that the deep learning network volume estimates would show stronger associations with cardiovascular risk factors and presence of CVD than standard volume estimates.

## Methods

### Data set

In this study, we used CMR cases from the UK Biobank under the terms of access approval number 2964. The UK Biobank is a population-based cohort study including over 500 000 participants aged 40–69 years, recruited between 2006 and 2010 (UK^10^). The CMR imaging study protocol was described previously.^11^ Briefly, CMR images were acquired on a 1.5 Tesla scanner (MAGNETOM Aera, Syngo Platform VD13A, Siemens Healthineers AG, Erlangen, Germany) utilizing retrospective electrocardiogram (ECG) gating. Short-axis cine images were acquired in a contiguous stack with slice thickness of 8 mm and spacing of 10 mm covering the LV and RV. Three long-axis cine slices were also acquired with 6 mm slice thickness, oriented in the four-chamber, two-chamber, and three-chamber views.

**Figure 1:**
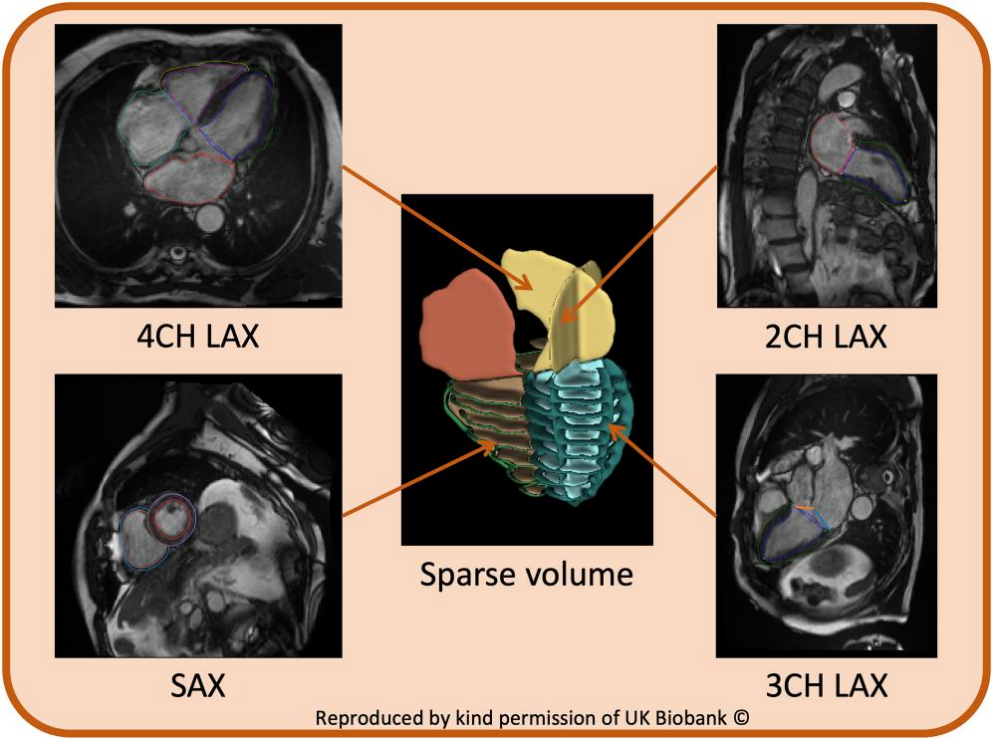
Example of cvi42 obtained LV endocardial, LV epicardial, and RV endocardial contours for short-axis as well as two-chamber, three-chamber, and four-chamber long-axis views. Contours are used to create a 3D sparse volume input. This figure contains images reproduced by kind permission of UK Biobank.

Short- and long-axis cine images were analysed automatically using Circle cvi42 version 5.11 release 1505 (Circle Imaging, Calgary, Canada). The software used a previously validated^12^ deep learning convolutional neural network and returned LV endocardial, LV epicardial, and RV endocardial contours for short-axis as well as three-chamber, two-chamber, and four-chamber long-axis views (*Figure 1*). Chamber volumes for the RV and LV and right and left atria were obtained from cvi42 reports, automatically generated from the machine learning contours. No manual correction was performed. ED and ES frame numbers were identified from the cvi42 reports.

Of the 45 683 participants with imaging studies available at the time of the study, we selected all participants with recorded CVD using hospital episode statistics and valid cvi42 reports. The following disease categories were defined based on codes ICD10, ICD9, and OPCS4: i) heart failure (HF); ii) myocardial infarction or ischaemic disease (MI-IHD); iii) ventricular arrhythmia composite (VAC) comprising ventricular arrhythmia or cardiac arrest or implantable cardiac defibrillator or sudden cardiac death; iv) conduction defect (CD) comprising bundle branch block or atrial or ventricular block; and v) atrial fibrillation (AF). We also selected diabetes mellitus (DM), which has significant comorbidity with CVD. These disease categories represent a wide range of pathologies known to have relationships with volume changes in the heart.^13^ Systolic and diastolic blood pressure was adjusted in the presence of blood pressure–altering medication (+15 mmHg and +10 mmHg, respectively) and averaged across multiple manual and automated readings. We also randomly selected a corresponding number of individuals with no recorded disease and valid cvi42 reports as a reference group.

Volumes were indexed to body surface area (BSA) using the Du Bois formula.^14,15^

### Quality assessment

We performed outlier rejection to remove cases with differences between network predicted and standard LV and RV ED and ES volumes and ejection fraction (EF) beyond the 3*interquartile range (IQR) of the first and third quartiles (less than Q1 − 3*IQR or greater than Q3 + 3*IQR).

### Deep learning network

**Figure 2:**
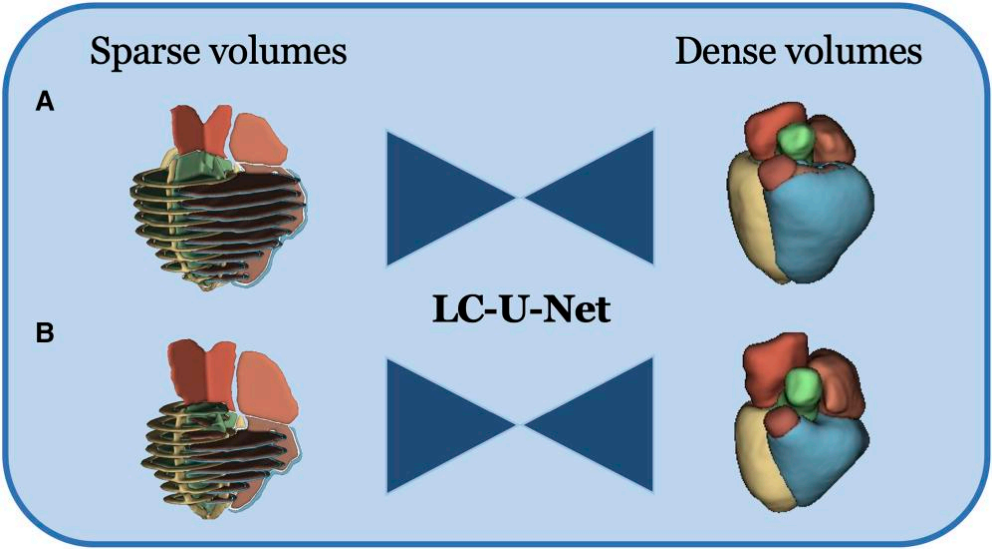
Method pipeline. Sparse volumes are fed into the LC-U-Net at inference time to reconstruct dense volumes. (*A*) ES frame. (*B*) ED frame.

The label completion network LC-U-Net described previously in Xu *et al.*^9^ was deployed for this study. Briefly, a 3D U-Net was designed to predict dense segmentation label maps (∼1 mm isotropic resolution) from sparse short- and long-axis slices. The input to the network is a 3D volume consisting of the sparse labels obtained from cvi42, whereas the CMR images are never used in the pipeline. The network reconstructs shapes in the missing slices and corrects for slice positioning errors and changes in breath-hold position between slices (*Figure 2*). Chamber volumes are then computed by summing the voxels of the dense segmentations and multiplying by the voxel volume.

The network was initially trained on sparse inputs obtained by 1400 coronary computed tomography data sets from the Scottish COmputed Tomography of the HEART (SCOT-HEART) trial,^16^ modified to resemble the Biobank MR data sets by simulating the placement of the short-axis and long-axis slices and adding random errors in slice positioning and breath-hold position to simulate motion. For this study, the pre-trained network was deployed on Biobank sparse label maps, obtained by combining short axes and two-, three-, and four-chamber long-axis slices, as automatically segmented by Circle (*Figure 1*).

### Statistics

To study the associations between volumes and risk factors, we considered three types of analysis:

- Univariate correlations between volumes and risk factors and disease [age, sex, systolic blood pressure, BSA, body mass index (BMI), AF, HF, MI-IHD, DM, CD, and VAC] were compared using *R*^2^ and −log(*P*). Note that −log(*P*) is used in PheWAS analyses in large cohort studies.^13^ The Bonferroni correction was applied, considering 88 numbers of individual tests (α* = 0.05/88).
- Multivariate linear regression was performed using all the aforementioned factors to predict volume in both ES and ED frames.
- Logistic regression was performed, considering the response variable positive if the estimated volume was above median value and negative if below. Predictor variables were the same as above. A five-fold cross validation was performed.

Regression models from the caret package v. 6.0^17^ in R 4.3.2^18^ were used.

Signed differences in volume were compared between the network and standard estimation from the Circle report files using paired *t*-test for normally distributed data, Wilcoxon rank sum test for non-normal data, and Pearson’s *χ*^2^ test for categorical data. Normality was tested using the *χ*^2^ test at a 95% confidence level. A *P-*value of 0.05 was considered significant. Area under the curve (AUC) for each logistic regression model (volume estimated from the network vs. standard estimates) was compared with the de Long test from the pROC package v.1.18.5.^19^

## Results

### Data set

A total of 4776 cases with CVD and 5795 randomly selected reference cases were included. After quality control (QC), we obtained a cohort of 4723 CVD cases and 5733 healthy individuals. The study population characteristics are summarized in *Table 1*. The characteristics of the cases rejected after quality control are reported in Supplementary data online, *Table S1*.

**Table 1:** Study population characteristics

	CVD	No CVD
	*n* = 4723	*n* = 5733
Age (years)	67 (7)	63 (8)
Male	3208 (68%)	2961 (52%)
Weight (kg)	83 (16)	77 (17)
Height (m)	171 (9)	170 (11)
BMI (kg/m^2^)	28.3 (4.6)	26.4 (4.2)
SBP (adjusted, mmHg)	147 (21)	138 (20)
DBP (adjusted, mmHg)	86 (12)	82 (11)
Atrial fibrillation (AF)	1011 (21%)	
Heart failure (HF)	339 (7.2%)	
MI or IHD	2212 (47%)	
DM	1490 (32%)	
Conduction defect (CD)	615 (13%)	
SCD, VA, or ICD	138 (2.9%)	

Continuous values are presented as ‘mean (standard deviation)’ and categorical values as ‘size (percentage)’. All rows *P* < 0.001 for difference between CVD and no-CVD groups.

BMI, body mass index; SBP, systolic blood pressure; DBP, diastolic blood pressure: MI, myocardial infarction; IHD, ischaemic heart disease; DM, diabetes mellitus; SCD, sudden cardiac death.

**Figure 3:**
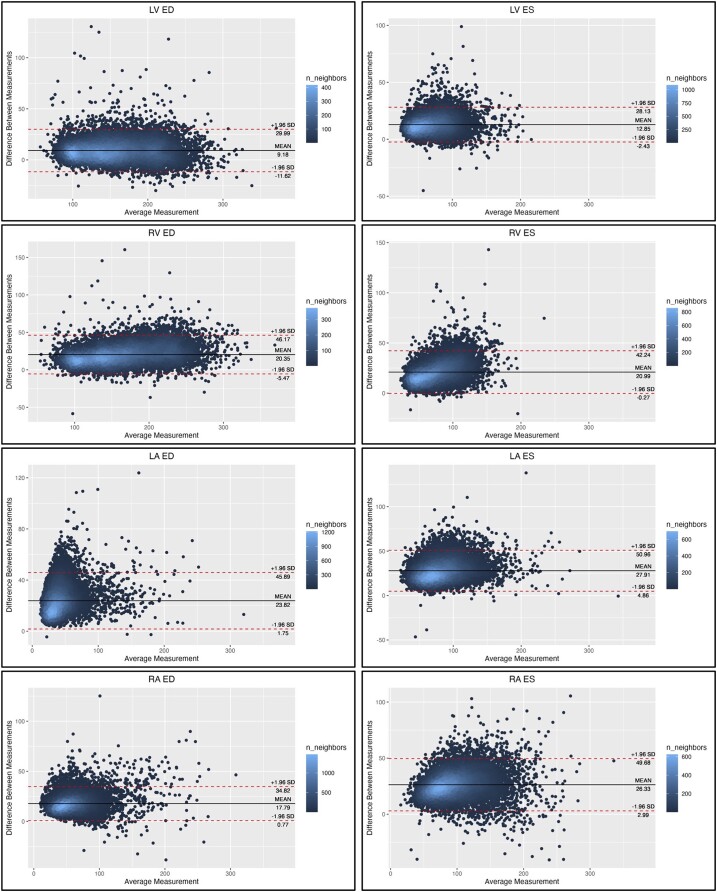
Bland–Altman plots for each volume to show difference between standard and network measurements. Each scatter point is coloured according to the number of neighbouring points (density map).

Volume estimates indexed by BSA from the network and standard methods are summarized in *Table 2* (see Supplementary data online, *Table S2*, for rejected cases). Bland–Altman plots are shown in *Figure 3*. The network predicted volumes were larger than the standard estimates for all chambers at both ED and ES. Both methods found significantly greater volumes in the CVD group than the no-CVD group for LV end-diastolic volume index (EDVI), RV EDVI, and RA end-systolic volume index (ESVI). LA EDVI was significantly smaller in the no-CVD group for both methods, but RA EDVI was smaller (*P* < 0.001) in the no-CVD group only for the network estimate. LA ESVI was significantly larger (*P* = 0.014) in the CVD group for standard method but not significantly different between groups for the network method.

**Table 2:** Volume indices estimated from the network and from standard methods

	Network		*P*-value	Standard		*P*-value
	CVD (*n* = 4723)	No CVD (*n* = 5733)		CVD (*n* = 4723)	No CVD (*n* = 5733)	
LV EDVI (mL/m^2^)	83 (16)	85 (19)	<0.001	78 (16)	81 (20)	<0.001
LV ESVI (mL/m^2^)	39 (10)	39 (11)	0.12	33 (11)	32 (11)	0.2
RV EDVI (mL/m^2^)	88 (16)	92 (23)	<0.001	77 (15)	82 (22)	<0.001
RV ESVI (mL/m^2^)	43 (10)	45 (14)	0.010	33 (9)	34 (12)	0.044
LA EDVI (mL/m^2^)	30 (13)	27 (8)	<0.001	17 (12)	14 (7)	<0.001
LA ESVI (mL/m^2^)	50 (14)	50 (12)	0.5	36 (14)	35 (12)	0.014
RA EDVI (mL/m^2^)	36 (15)	34 (11)	<0.001	26 (14)	25 (11)	0.3
RA ESVI (mL/m^2^)	57 (16)	59 (17)	<0.001	43 (16)	45 (16)	<0.001

*P*-value shown between CVD and no-CVD group for both methods.

### Univariate associations

**Figure 4:**
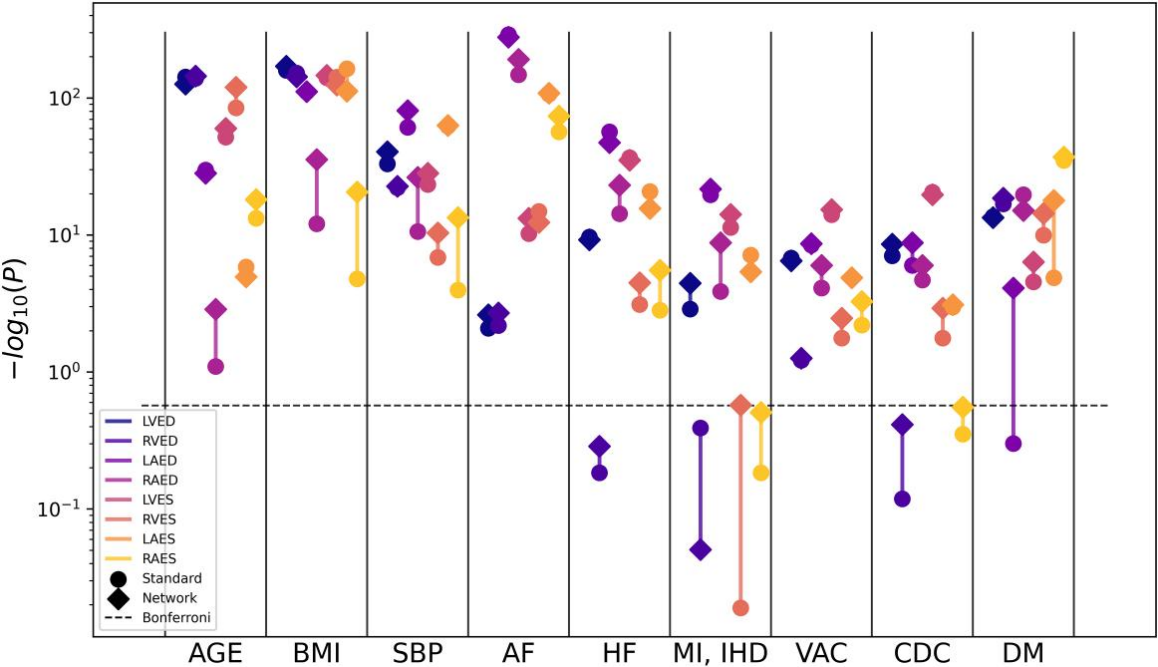
Manhattan plot showing the strength of correlation between volumes and single predictors. The height of each data point denotes the negative logarithm of the univariate correlation *P-*value between each volume and predictor. The Bonferroni threshold for multiple comparisons (*α* = 0.05/88) is shown as a dashed horizontal line. Sex and BSA have been removed for visualization purposes (−logp = inf). Results are included in Supplementary data online, *Table S3*. Log scale is applied to *y*-axis.

*Figure 4* compares the univariate association of volumes with risk and disease factors between network and standard estimations. The *x*-axis shows each individual predictor, and the *y*-axis shows the strength of correlation found between each factor and the volume values. In most comparisons, the −log(*P*) was bigger for network estimated volumes compared to standard estimated volumes, suggesting stronger univariate associations. In all associations between network estimated volumes and age and BSA, the *P-*value was 0 to machine precision. The quantitative results can be found in Supplementary data online, *Table S3*.

### Multivariate associations

**Figure 5:**
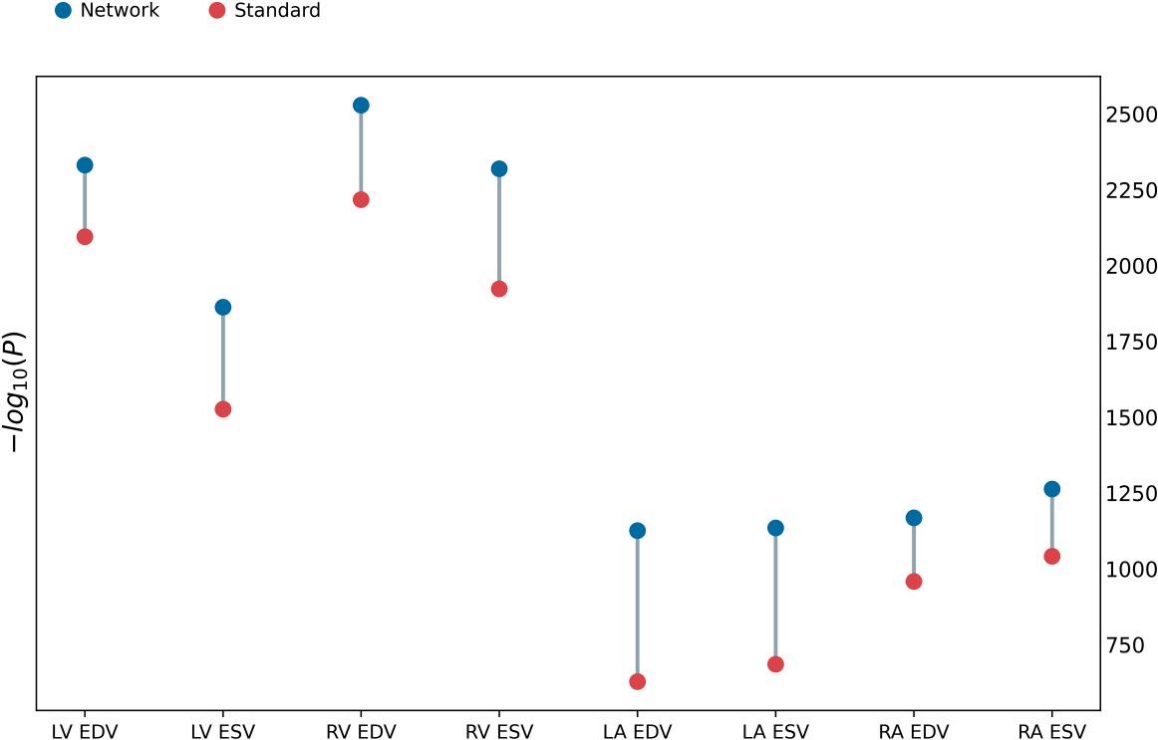
Dumbbell chart of −log(*P*) of the overall multivariate regression model (overall cases in both groups).

The Dumbbell chart in *Figure 5* shows an overview of the multivariate regression model results, by plotting the −log(*P*) values for each volume. Detailed results of the multivariate regression are shown in *Table 3*. The *R*^2^, −log(*P*), and *F*-values show overall regression statistics, which demonstrate a higher overall correlation between the network estimated volumes and the risks and disease factors than for the standard estimated ones for all chambers. Most predictors in *Table 3* show higher −log(*P*) values for the network over the standard volumes, indicating stronger relationships.

**Table 3:** Results of multivariate regressions

	LV EDV	LV ESV	RV EDV	RV ESV	LA EDV	LA ESV	RA EDV	RA ESV
*R* ^2^	**0.64**/0.61	**0.56**/0.49	**0.67**/0.63	**0.64**/0.57	**0.39**/0.25	**0.40**/0.26	**0.41**/0.35	**0.43**/0.37
−log(*P*)	**2331**/2095	**1863**/1525	**2529**/2217	**2319**/1923	**1126**/628	**1135**/685	**1168**/958	**1263**/1041
*F*	**1722**/1457	**1863**/1526	**1965**/1590	**1708**/1282	**620**/310	**626**/342	**649**/507	**718**/562
Age	151/*158*	**51**/37	**155**/127	**112**/49	**53**/22	1/*5*	**15**/11	**3**/0
Sex	**202**/136	**180**/136	**297**/208	**Inf**/256	**32**/4	**27**/7	**77**/74	74/74
BSA	Inf/Inf	**Inf**/253	Inf/Inf	**Inf**/297	**176**/54	**255**/111	**201**/154	**282**/213
BMI	52/*53*	**33**/19	**64**/46	**48**/18	**11**/2	**27**/0	60/*67*	113/*115*
SBP (adj)	**23**/19	**2**/1	7/7	1/*7*	**8**/6	20/20	0/*2*	1/1
AF	5/5	**1**/0	2/*3*	4/*6*	**242**/233	**86**/81	**174**/126	**61**/42
HF	11/*13*	**37**/36	0/0	**6**/3	22/*24*	7/*9*	**10**/6	**3**/2
MI + IHD	7/7	2/*3*	23/*24*	22/*26*	**1**/0	**4**/1	5/*8*	12/*15*
DM	**88**/76	**45**/33	**105**/90	**72**/55	**30**/12	**62**/32	**34**/29	**62**/43
VA	4/4	**7**/5	0/0	0/0	0/*1*	1/1	0/0	**1**/0
CD	**10**/8	15/15	0/0	**2**/1	1/*2*	**1**/0	0/0	**2**/1

*R* ^2^, −log(*P*), and *F* show overall regression statistics. Individual predictors show −log(*P*) for multivariate model. All results are network/standard. Events where the network has a higher value are highlighted in bold; the opposites are highlighted in italics.

### Logistic regressions

**Figure 6:**
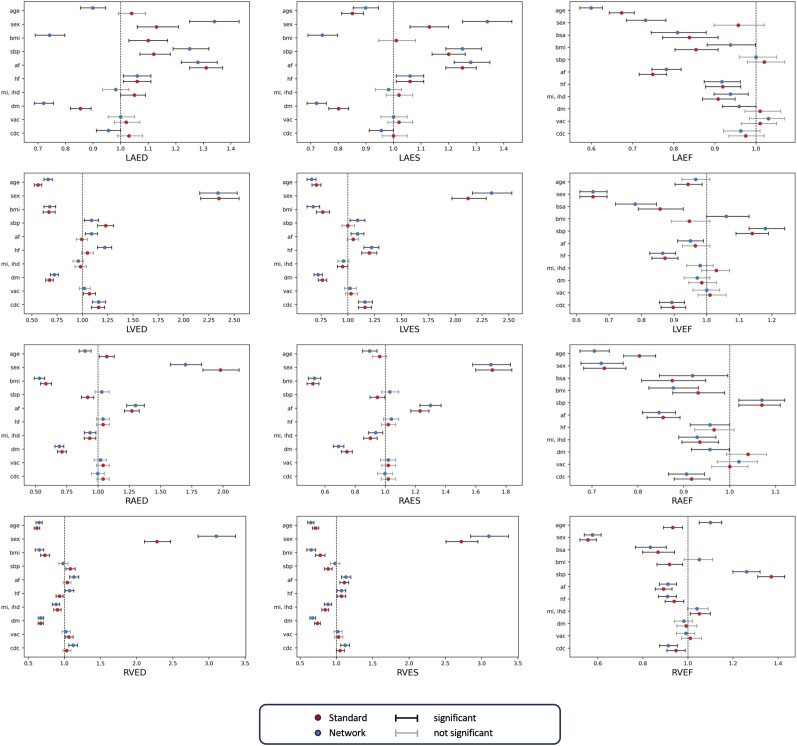
Odds ratios for comparison between results of the logistic regression model using estimate volumes by standard and network methods. The BSA predictor in ED and ES volumes has been removed for visualization purposes (see Supplementary data online, *Table S4*, for complete results).

*Table 4* and *Figure 6* show the results of the logistic regression. *Table 4* is a summary of the AUC values achieved by the two logistic regression models trained with the network and the standard estimations, respectively. Network volumes had significantly higher AUC for every chamber at ES and ED. *Figure 6* shows the relevant difference between each individual predictor in the regression through odds ratios. The numerical results are reported in Supplementary data online, *Table S4*. These results highlight some significant results in the logistic regression for the network predicted volumes that are not found in the standard ones, especially for the LA ED and ES volumes and for most EF plots.

**Table 4:** Results of logistic regressions

	AUC (network)	AUC (standard)	*P*-value
LV EDV	0.918 [0.912–0.923]	0.902 [0.897–0.908]	<0.001
LV ESV	0.909 [0.903–0.915]	0.885 [0.878–0.891]	<0.0001
RV EDV	0.928 [0.923–0.932]	0.910 [0.904–0.915]	<0.0001
RV ESV	0.923 [0.918–0.928]	0.904 [0.898–0.910]	<0.0001
LA EDV	0.862 [0.855–0.869]	0.733 [0.723–0.742]	<0.0001
LA ESV	0.829 [0.821–0.837]	0.757 [0.748–0.766]	<0.0001
RA EDV	0.877 [0.870–0.884]	0.845 [0.834–0.853]	<0.0001
RA ESV	0.852 [0.844–0.859]	0.829 [0.821–0.834]	<0.0001

AUC shows overall test set results and *P*-value from de Long test; 95% confidence intervals are included in square brackets.

## Discussion

In this study, we evaluated an automated method for whole heart chamber volume quantification from short- and long-axis slices, which are typically acquired in all CMR exams. Heart volumes are currently computed from CMR slices using geometric assumptions, including regular ellipses for the atria and slice summation for the ventricles.^3^ The assumptions may lead to inaccuracies; for example, apical and basal anatomy is not well captured by short-axis slices,^20^ which are typically much thicker than the in-plane resolution (6–8 mm vs. 1–2 mm). Advances in deep learning networks enable accurate reconstruction of 3D anatomy from sparse data, as demonstrated in our previous paper,^9^ where we introduced and validated a neural network (LC-U-Net) to reconstruct dense volumes from CMR slices. On a test set including 200 samples from SCOT-HEART, we found signed differences in volume between network predictions and ground truth segmentations of 0.4 ± 2.6 mL for LV volume, 2.7 ± 4.4 mL for RV volume, −1.0 ± 4.0 mL for LA volume, and 1.3 ± 6.3 mL for RA volume. The promising performance led us to investigate the effect that LC-U-Net predictions have on volumes by calculating associations with risk factors and using a large cohort study such as the UK Biobank.

In the present work, we employed the pre-trained model validated on SCOT-HEART data to predict 20 890 3D heart volumes, including both healthy and diseased individuals during ED and ES.

To ensure that the network was robust against common CMR artefacts and acquisition, during training on the original data, motion artefact was simulated by a 2D in-plane translation of the heart with three different levels of Gaussian standard deviation for offsets in each direction: tiny (0.5 mm), small (2.0 mm), and standard (3.5 mm). Uniaxial motion was applied in all three axes, and, for each slice, one set of motions was applied to the reference shape and its intersection with the slice plane was considered as the simulated segmentation of the slice. Moreover, the most apical and basal short-axis (SAX) slices were removed (probability 0.5) and labelled PA as RV in the input SAX slice (if present).^9^

We performed univariate, multivariate, and logistic regression on those to establish correlations with the risk and disease factors, and we compared the results with the same analysis performed on volumes from a standard automatic pipeline by the widely available software cvi42.

We found that, when evaluating the volumes, the network consistently predicted larger values than the standard method. We believe this to be due to inherent limitations of the mathematical formulae used in standard methods. The bi-planar approach adopted often leads to underestimation of volumes, especially in the LA,^8,21^ while in the ventricles, the outflow tracts are difficult to quantify in short-axis images.^22^

Our results also showed that network estimates may contain more diagnostic information over traditional estimates. The multivariate linear regression results showed that all chamber volumes had higher correlations with risk and disease factors when obtained from network predictions, with the highest benefit demonstrated for LA volumes (*Figure 5*).

Correlations between single factors and volumes were shown by the univariate and logistic regression results. The former found stronger associations between (i) ventricle volumes at ES with age and BMI and (ii) atrial volumes at ED with AF and systolic blood pressure, when using network estimations. The standard estimations unveiled higher correlation between LA volumes at ES and BMI (*Figure 4*, Supplementary data online, *Table S3*). The logistic regression highlighted correlations between EF in the atria and DM and between LV measures and AF, which showed more significant odds ratios than in the standard counterparts (*Figure 6*, Supplementary data online, *Table S4*). This supports the use of network estimates to quantify cardiac structure more accurately in the evaluation of disease. Also, relationships with sex and DM were stronger in the network predicted metrics than the traditional in all chambers, indicating the benefit of network estimates in cohort studies with large patient data. We therefore expect that clinical studies of sex differences in diabetes would need fewer participants to demonstrate a target effect size. To illustrate this, in *Table 5*, we show differences between men and women in the diabetic subcohort. The sex differences are generally greater for the network estimates than the standard estimates in this group. For example, a power calculation of the number of participants needed for a study of sex differences in LV EDVI in diabetes would need 38 men and 38 women for the network estimation method (80 vs. 71 with the higher standard deviation seen in men of 14 mL/m^2^) and 72 men and 72 women for the standard estimation method (68 vs. 75 with the higher standard deviation seen in men of 15 mL/m^2^) to detect a difference at alpha of 5% and 80% power.

**Table 5:** Differences between men and women for participants with diabetes

	Network		*P*-value	Standard		*P*-value
	Female (*n* = 533)	Male (*n* = 937)		Female (*n* = 533)	Male (*n* = 937)	
LV EDVI (mL/m^2^)	71 (11)	80 (14)	<0.001	68 (12)	75 (15)	<0.001
LV ESVI (mL/m^2^)	32 (7)	39 (10)	<0.001	26 (7)	32 (10)	<0.001
RV EDVI (mL/m^2^)	74 (12)	86 (14)	<0.001	67 (12)	75 (14)	<0.001
RV ESVI (mL/m^2^)	34 (7)	43 (9)	<0.001	26 (6)	33 (8)	<0.001
LA EDVI (mL/m^2^)	23 (7)	27 (11)	<0.001	14 (7)	16 (11)	0.13
LA ESVI (mL/m^2^)	43 (10)	46 (13)	<0.001	33 (10)	33 (12)	0.8
RA EDVI (mL/m^2^)	27 (7)	33 (12)	<0.001	18 (7)	24 (12)	<0.001
RA ESVI (mL/m^2^)	46 (11)	52 (14)	<0.001	33 (10)	40 (14)	<0.001

Overall, we found stronger associations between heart chamber volumes and CVD and risk factors using network estimates than with standard volume estimates. The ability to more readily distinguish effect sizes enables study of disease interactions with higher precision. These results support automated computation of volumes using deep learning networks, which can be performed in routine CMR exams.

### Limitations and future work

The main limitation of this work lies in the lack of 3D ground truth for volumes in the UK Biobank, so the reconstructed shapes by the LC-U-Net could not be validated. However, the network was previously validated in a test set from the SCOT-HEART CT study against 3D ground truth segmentations. Also, we did not attempt to propensity match or stratify reference cases with disease cases in this study, or evaluate comorbidities, since our objective was to compare deep learning and standard volumes in a diverse cohort rather than identify specific disease characteristics. Future study with more disease cases could identify relationships disentangled from comorbidities. In this study, we rejected cases with high differences between network predicted and standard LVED, RVED, LVES, RVES, and EF. We observed the rejected cases would often include examples with large ventricles (see Supplementary data online, *Figure S1*), showing that the network might need further training on such cases to guarantee better adaptation. Supplementary data online, *Figure S1*, shows an example of a rejected case in which the prediction was obviously smaller than the slice data. To ensure that this would not compromise the performance of our regression experiments, we further tested consistency between groups with low and high EFs or groups with small and high LVEDV by stratifying the cohort based on median values and performing the multivariate regression analysis as in the main study. The results can be found in Supplementary data online, *Tables S5–S8*, and they confirm the findings of higher correlation in the network estimated volumes for all subgroups.

Furthermore, the contours obtained from cvi42 could undergo further QC as in Ruijsink *et al.*,^23^ which, if done, could possibly boost the results of the regression models for both standard predicted volumes and network predicted. Finally, the availability of certain disease cohorts is limited in UK Biobank, since it comprises largely healthy participants. Further validation of these methods in disease cohorts is required. This could be performed by applying network and standard methods to repeated scans and estimate and compare variance (follow-up exams are underway as part of the UK Biobank imaging substudy). Similarly, it would be possible to apply the reconstruction throughout the cardiac cycle to obtain full dense segmentations at every acquired frame. The 3D segmentations could also be used as an updated ground truth for training networks to make automatic segmentations more reliable. These could also lead to further studies on 3D strain and shape analysis, which were not considered here.

## Conclusion

Stronger relationships between heart chamber volumes and disease and risk factors were obtained using a deep learning volume reconstruction compared with standard volume estimates, in the CMR UK Biobank study. Deep learning estimates of volumes may provide more information for diagnosis and prognosis than standard estimates.

## Supplementary Material

jeae123_Supplementary_Data

## Data Availability

Code and trained weights for the deep learning network are available from cemrg.com. Volume reconstructions will be made available through the UK Biobank data return mechanism (access 2964). The UK Biobank makes all data available to all bona fide researchers for all types of health-related research that is in the public interest, without preferential or exclusive access for any person (please see the UK Biobank’s website for the detailed access procedure: http://www.ukbiobank.ac.uk/register-apply/).
