# Supplementary material for: Evaluation of deep learning estimation of whole heart anatomy from automated cardiovascular magnetic resonance short- and long-axis analyses in UK Biobank
Source: Eur Heart J Cardiovasc Imaging. 2024 May 9;25(10):1374–83. doi: 10.1093/ehjci/jeae123 (PMC11441036; doi:10.1093/ehjci/jeae123)
Supplement: jeae123_Supplementary_Data [file jeae123_supplementary_data.docx]

**Supplementary**

Fig. S1: Example of rejected case. A) sparse label map, B) overlayed dense prediction by the network on sparse label map.


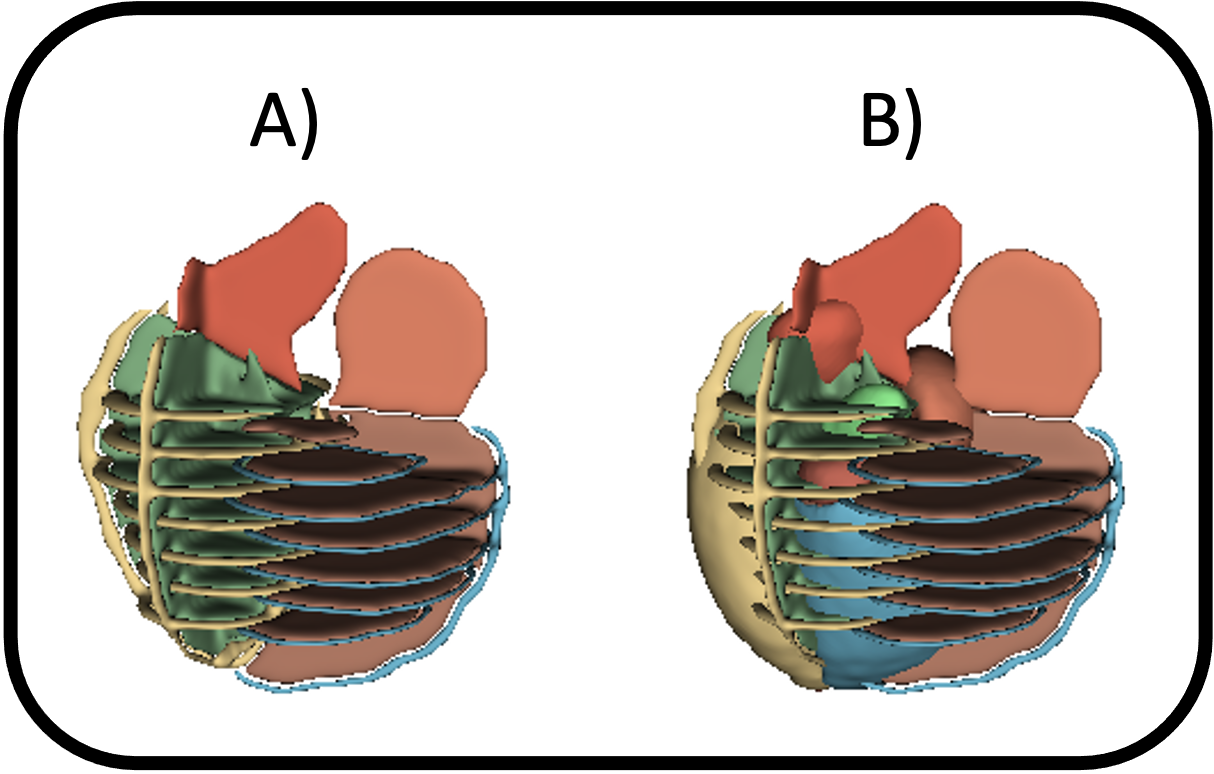


Table S1: Study population characteristics of the rejected cases. Continuous values are presented as “mean (standard deviation)”, while categorical values as “size (percentage)”. BMI: body mass index; SBP: systolic blood pressure; DBP: diastolic blood pressure: MI: myocardial infarction; IHD: ischemic heart disease; DM: Diabetes mellitus; SCD: sudden cardiac death. * = p<0.05 for difference between CVD and no CVD groups.

|  | CVD  n = 53 | No CVD  n = 62 |
| --- | --- | --- |
| Age (years) | 67 (7) | 64 (8) * |
| Male | 47 (89%) | 45 (73%) * |
| Weight (kg) | 92 (17) | 88 (20) |
| Height (m) | 175 (7) | 176 (12) |
| BMI (kg m^-2^) | 29.7 (4.5) | 28.2 (4.9) |
| SBP (adjusted, mmHg) | 149 (18) | 144 (22) |
| DBP (adjusted, mmHg) | 87 (11) | 85 (12) |
| Atrial Fibrillation | 11 (21%) | - |
| Heart Failure | 5 (9.4%) | - |
| MI or IHD | 25 (47%) | - |
| DM | 16 (30%) | - |
| Conduction defect | 13 (25%) | - |
| SCD or VA or ICD | 5 (9.5%) |  |

Table S2: Volume indices estimated from the network and from standard methods in the rejected cases. P-Value between CVD and no CVD group for significance.

|  | **Network** | | **P-Value** | **Standard** | | **P-Value** |
| --- | --- | --- | --- | --- | --- | --- |
|  | CVD  (n = 53) | No CVD  (n = 62) |  | CVD  (n = 53) | No CVD  (n = 62) |  |
| **LV EDVI (ml/m^2^)** | 93 (20) | 95 (21) | 0.4 | 76 (25) | 73 (26) | 0.8 |
| **LV ESVI (ml/m^2^)** | 52 (15) | 51 (14) | >0.9 | 33 (16) | 35 (17) | 0.5 |
| **RV EDVI (ml/m^2^)** | 95 (22) | 102 (26) | 0.13 | 73 (23) | 74 (28) | 0.7 |
| **RV ESVI (ml/m^2^)** | 57 (17) | 59 (18) | 0.7 | 36 (16) | 36 (15) | 0.8 |
| **LA EDVI (ml/m^2^)** | 39 (20) | 32 (9) | 0.14 | 22 (20) | 16 (8) | 0.2 |
| **LA ESVI (ml/m^2^)** | 56 (22) | 53 (15) | >0.9 | 40 (20) | 37 (14) | >0.9 |
| **RA EDVI (ml/m^2^)** | 41 (21) | 38 (12) | 0.3 | 31 (21) | 28 (12) | 0.6 |
| **RA ESVI (ml/m^2^)** | 59 (22) | 60 (20) | 0.8 | 44 (20) | 45 (18) | 0.7 |

Table S3: Results of Univariate Regressions (-log(p)). All results are network/standard. Inf: p value is 0 to machine precision. Events where the network has a higher value are highlighted in bold green, the opposites are highlighted in bold red.

|  | **LV EDV** | **LV ESV** | **RV EDV** | **RV ESV** | **LA EDV** | **LA ESV** | **RA EDV** | **RA ESV** |
| --- | --- | --- | --- | --- | --- | --- | --- | --- |
| **Age** | 126/**142** | **59**/52 | **144**/139 | **120**/85 | 28/**30** | 5/**6** | **3**/1 | **18**/13 |
| **Sex** | Inf/Inf | Inf/Inf | Inf/Inf | Inf/Inf | **Inf**/175 | **Inf**/316 | Inf/Inf | Inf/Inf |
| **BSA** | Inf/Inf | Inf/Inf | Inf/Inf | Inf/Inf | **Inf**/283 | Inf/Inf | Inf/Inf | Inf/Inf |
| **BMI** | **170**/159 | **146**/141 | 143/**152** | 124/**142** | 111/111 | 112/**164** | **36**/12 | **21**/5 |
| **SBP (adj)** | **40**/33 | **28**/23 | **23**/22 | **10**/7 | **81**/61 | **63**/62 | **26**/11 | **13**/4 |
| **AF** | **3**/2 | **13**/10 | **3**/2 | 12/**15** | 277/**290** | **108**/107 | **191**/148 | **74**/56 |
| **HF** | 9/**10** | 35/**37** | 0/0 | **4**/3 | 47/**57** | 16/**21** | **23/**14 | **6**/3 |
| **MI+IHD** | **4**/3 | **14**/11 | 0/0 | 1/0 | **22**/20 | 5/**7** | **9**/4 | **1**/0 |
| **VA** | 6/**7** | **15**/14 | 1/1 | 2/2 | **9**/8 | 5/5 | **6**/4 | **3**/2 |
| **CD** | **9**/7 | 20/20 | 0/0 | **3**/2 | **9**/6 | 3/3 | **6**/5 | **3**/0 |
| **DM** | 13/**14** | **6**/5 | **19**/17 | **14**/10 | **4**/0 | **18**/5 | 15/**20** | **37**/35 |

Table S4: Results of Logistic Regressions. AUC show overall test set results (p from de Long test, *p<0.001). Individual predictors show Odds Ratios for multivariate model. All results are network/standard. Odds ratios where the network had a value further from 1.0 are highlighted in bold green, the opposites are highlighted in bold red.

|  | **LV EDV** | **LV ESV** | **RV EDV** | **RV ESV** | **LA EDV** | **LA ESV** | **RA EDV** | **RA ESV** |
| --- | --- | --- | --- | --- | --- | --- | --- | --- |
| **AUC (LR)** | **0.92**/0.90 | **0.91**/0.89 | **0.93**/0.91 | **0.92**/0.90 | **0.86**/0.73 | **0.83**/0.76 | **0.88**/0.85 | **0.85**/0.83 |
| **Age** | 0.6/0.6 | 0.7/0.7 | 0.6/0.6 | **0.6**/0.7 | **1.2**/1.0 | 0.9/0.9 | 1.1/1.1 | **0.9**/1.0 |
| **Sex** | **2.4**/2.0 | **2.3**/2.1 | **3.1**/2.3 | **3.1**/2.7 | **1.6**/1.2 | **1.3**/1.1 | 2.0/2.0 | 1.7/1.7 |
| **BSA** | **6.7**/6.2 | **5.8**/4.2 | **6.4**/5.9 | **5.8**/4.6 | **4.5**/2.0 | **3.8**/2.4 | **4.7**/3.4 | **4.2**/3.5 |
| **BMI** | 0.7/0.7 | **0.7**/0.8 | 0.7/0.7 | **0.7**/0.8 | **0.7**/1.1 | **0.7**/1.0 | 0.6/0.6 | 0.5/0.5 |
| **SBP (adj)** | 1.2/1.2 | **1.1**/1.0 | 1.1/1.1 | 1.0/**0.9** | **1.2**/1.1 | **1.3**/1.2 | 1.0/**0.9** | 1.0/**0.9** |
| **AF** | 1.0/1.0 | 1.1/1.1 | **1.1**/1.0 | 1.1/1.1 | **1.4**/1.3 | 1.3/1.3 | **1.4**/1.3 | **1.3**/1.2 |
| **HF** | 1.1/1.1 | 1.2/1.2 | 1.0/**0.9** | 1.1/1.1 | 1.1/1.1 | 1.1/1.1 | 1.0/1.0 | 1.0/1.0 |
| **MI+IHD** | 1.0/1.0 | 1.0/1.0 | 0.9/0.9 | 0.9/**0.8** | 1.0/**1.1** | 1.0/1.0 | 1.0/**0.9** | 0.9/0.9 |
| **DM** | 0.7/0.7 | **0.7**/0.8 | 0.7/0.7 | 0.7/0.7 | **0.7**/0.9 | **0.7**/0.8 | 0.7/0.7 | 0.7/0.7 |
| **VA** | **1.1**/1.0 | 1.0/1.0 | 1.0/**1.1** | 1.0/1.0 | 1.0/1.0 | 1.0/1.0 | 1.0/1.0 | 1.0/1.0 |
| **CD** | **1.2**/1.1 | 1.2/1.2 | 1.0/1.0 | 1.1/1.1 | 1.0/1.0 | 1.0/1.0 | 1.0/1.0 | 1.0/1.0 |

Table S5: Results of Multivariate Regressions in high Ejection Fraction cohort. R2, -log(p) and F show overall regression statistics. Individual predictors show -log(p) for multivariate model. All results are network/standard. Events where the network has a higher value are highlighted in bold green, the opposites are highlighted in bold red.

|  | **LV EDV** | **LV ESV** | **RV EDV** | **RV ESV** | **LA EDV** | **LA ESV** | **RA EDV** | **RA ESV** |
| --- | --- | --- | --- | --- | --- | --- | --- | --- |
| **R2** | **0.71**/0.66 | **0.69**/0.62 | **0.72**/0.66 | **0.7**/0.61 | **0.46/**0.21 | **0.45**/0.29 | **0.51**/0.4 | **0.49**/0.41 |
| **-log(p)** | **1378**/1220 | **1331**/1093 | **1435**/1196 | **1355**/1064 | **684**/258 | **658**/369 | **799**/570 | **746**/584 |
| **F** | **1145**/934 | **1079**/785 | **1229**/905 | **1113**/753 | **403**/127 | **383**/189 | **497**/319 | **452**/329 |
| **Age** | 101/**104** | **91**/84 | **80**/61 | **86**/36 | **6**/2 | 9/**13** | 0/**2** | **7**/1 |
| **Sex** | **125**/73 | **128**/74 | **178**/107 | **191**/130 | **30**/3 | **22**/4 | **71**/59 | **50**/43 |
| **BSA** | **317**/299 | **297**/243 | **308**/259 | **254**/180 | **134**/34 | **148**/65 | **167**/101 | **176**/129 |
| **BMI** | 30/**34** | **29**/26 | **30**/24 | **24**/9 | **7**/3 | **13**/1 | 41/**42** | 61/**66** |
| **SBP (adj)** | **10**/8 | **3**/2 | 1/**2** | 1/**5** | **8**/4 | 9/9 | 1/**2** | 0/**1** |
| **AF** | **1**/0 | 0/0 | 0/0 | 0/0 | 28/**38** | 11/**12** | **12**/10 | **6**/4 |
| **HF** | **1/**0 | **1**/0 | 0**/1** | 0/0 | **4**/2 | **2**/1 | **1**/0 | **1**/0 |
| **MI+IHD** | **5**/3 | 4/4 | **9**/7 | **9**/7 | 0/**3** | 1/1 | 1/**2** | 3/**5** |
| **DM** | **35**/29 | **34**/28 | **36**/30 | **33**/24 | **23**/9 | **26**/13 | **21**/16 | **26**/18 |
| **VA** | 0/0 | 0/0 | 0/0 | 0/0 | 1/1 | **1**/0 | 0/0 | 0/0 |
| **CD** | 2/2 | 1/**2** | 0/0 | 0/0 | 0/**1** | 0/**1** | 0/0 | 0/0 |

Table S6: Results of Multivariate Regressions low Ejection Fraction cohort. R2, -log(p) and F show overall regression statistics. Individual predictors show -log(p) for multivariate model. All results are network/standard. Events where the network has a higher value are highlighted in bold green, the opposites are highlighted in bold red.

|  | **LV EDV** | **LV ESV** | **RV EDV** | **RV ESV** | **LA EDV** | **LA ESV** | **RA EDV** | **RA ESV** |
| --- | --- | --- | --- | --- | --- | --- | --- | --- |
| **R2** | **0.55**/0.52 | **0.46**/0.43 | **0.6**/0.57 | **0.56**/0.5 | **0.37**/0.28 | **0.35**/0.25 | **0.35**/0.31 | **0.37**/0.32 |
| **-log(p)** | **898**/830 | **692**/619 | **1024**/948 | **927**/781 | **519**/358 | **478**/312 | **485**/416 | **511**/431 |
| **F** | **585**/524 | **409**/354 | **710**/634 | **613**/482 | **284**/183 | **256**/157 | **261**/217 | **278**/227 |
| **Age** | 62/**65** | **17**/15 | **78**/67 | **54**/25 | **35**/20 | **2**/0 | **15**/9 | 0/0 |
| **Sex** | **77**/59 | **58**/48 | **119**/98 | **125**/105 | **6**/1 | **8**/3 | 20/**22** | 27/**30** |
| **BSA** | **197**/186 | **142**/123 | **215/**198 | **174**/135 | **80**/27 | **120**/51 | **87**/72 | **124**/93 |
| **BMI** | **24**/23 | **14**/12 | **36**/24 | **27**/13 | **7**/0 | **17**/0 | 32/**37** | **59**/56 |
| **SBP (adj)** | **15**/14 | **6**/5 | **7**/6 | 1/1 | **8**/6 | **14**/13 | **1**/0 | **2**/0 |
| **AF** | **11**/9 | 0/**1** | **4**/3 | 4/**7** | **204**/185 | **78**/72 | **157**/117 | **59**/42 |
| **HF** | 9**/13** | 21/**24** | 0/**1** | 2/2 | 9/**14** | 4/**7** | 4/4 | 2/2 |
| **MI+IHD** | 3/**4** | 1/1 | 15/**19** | 17/**19** | **3**/1 | **4**/2 | 6/6 | **11**/10 |
| **DM** | **54**/47 | **30**/22 | **70**/62 | **50**/36 | **16**/6 | **38**/20 | **20**/16 | **38**/26 |
| **VA** | 4/**6** | **9**/8 | 0/**1** | 0/0 | 0/0 | 0/**1** | 0/0 | **1**/0 |
| **CD** | **7**/4 | 7/7 | 0/0 | **1**/0 | 2/**3** | 1/1 | 1/1 | 2/2 |

Table S7: Results of Multivariate Regressions in high LVED cohort. R2, -log(p) and F show overall regression statistics. Individual predictors show -log(p) for multivariate model. All results are network/standard. Events where the network has a higher value are highlighted in bold green, the opposites are highlighted in bold red.

|  | **LV EDV** | **LV ESV** | **RV EDV** | **RV ESV** | **LA EDV** | **LA ESV** | **RA EDV** | **RA ESV** |
| --- | --- | --- | --- | --- | --- | --- | --- | --- |
| **R2** | 0.26/0.26 | **0.15/**0.14 | **0.38**/0.35 | **0.3**/0.26 | **0.2**/0.16 | **0.13**/0.1 | **0.16**/0.15 | 0.16/0.16 |
| **-log(p)** | **332**/325 | **169**/159 | **532**/477 | **392**/337 | **241**/193 | **147**/105 | **193**/179 | **193**/188 |
| **F** | **168**/164 | **81**/76 | **292**/256 | **203**/171 | **118**/93 | **70**/50 | **93**/86 | **93/**90 |
| **Age** | 30/**32** | **2/**1 | **55**/42 | **36**/12 | **46**/30 | **10**/3 | **21**/14 | 2/2 |
| **Sex** | **28**/26 | 17/**25** | **69**/57 | 67/**68** | **2**/0 | **1**/0 | 13/**21** | 12/**19** |
| **BSA** | **98**/91 | **53**/36 | **122**/103 | **84**/60 | **43**/10 | **45**/14 | **49**/36 | **55**/39 |
| **BMI** | **14**/13 | **7**/3 | **27**/17 | **17**/6 | 3/**4** | **6**/5 | 25/**29** | 45/**48** |
| **SBP (adj)** | 8/8 | 0/0 | 1/**2** | 4/**10** | **6**/5 | 11/**12** | 0/**2** | 0/**2** |
| **AF** | **11**/10 | 2/**3** | 8/8 | 0/0 | **89**/87 | **29**/26 | **58**/39 | **19**/10 |
| **HF** | **14**/13 | **32**/30 | 0/0 | **4**/1 | 13/**14** | 4/**5** | **6**/4 | **2**/1 |
| **MI+IHD** | **23**/18 | **5**/4 | **51**/46 | 31/31 | **5**/1 | **15**/4 | 10/**11** | **21**/19 |
| **DM** | **41**/35 | **14**/9 | **57**/49 | **34**/28 | **11**/5 | **25**/13 | **11**/9 | **24**/15 |
| **VA** | 3/**4** | 5/5 | 0/0 | 0/0 | 1/1 | 1/1 | **1**/0 | **1**/0 |
| **CD** | 1/1 | 3/**4** | 4/4 | 0/0 | 2/2 | **4**/1 | **2**/1 | **6**/4 |

Table S8: Results of Multivariate Regressions in low LVED cohort. R2, -log(p) and F show overall regression statistics. Individual predictors show -log(p) for multivariate model. All results are network/standard. Events where the network has a higher value are highlighted in bold green, the opposites are highlighted in bold red.

|  | **LV EDV** | **LV ESV** | **RV EDV** | **RV ESV** | **LA EDV** | **LA ESV** | **RA EDV** | **RA ESV** |
| --- | --- | --- | --- | --- | --- | --- | --- | --- |
| **R2** | **0.42**/0.36 | **0.42**/0.32 | **0.43**/0.38 | **0.48**/0.41 | **0.35**/0.24 | **0.22**/0.16 | **0.34**/0.3 | 0.25/0.25 |
| **-log(p)** | **598**/490 | **611**/425 | **632**/536 | **719**/594 | **473**/295 | **272**/184 | **458**/399 | 316/**318** |
| **F** | **338**/264 | **348**/223 | **363**/295 | **430**/336 | **253**/147 | **134**/88 | **243**/207 | 159/**160** |
| **Age** | 63/**69** | **20**/14 | **29**/20 | **18**/4 | **34**/11 | **3/**1 | **17**/13 | 4/**6** |
| **Sex** | **10**/3 | **42**/28 | **49**/32 | **110**/87 | **3**/0 | **7**/6 | 17/**19** | 1/**8** |
| **BSA** | **204**/165 | **146**/80 | **169**/133 | **136**/86 | **45**/9 | **63**/17 | **66**/53 | **95**/80 |
| **BMI** | 19/**20** | **12**/4 | **14**/7 | **12**/1 | **3**/2 | **8**/1 | 22/**26** | 50/**52** |
| **SBP (adj)** | 5/5 | 1/**2** | 0/0 | 7/**10** | 1/1 | 2/**3** | 2/**3** | 2/**3** |
| **AF** | 4/4 | **27**/20 | **8**/7 | **35**/31 | **221**/192 | **118**/99 | **173**/137 | **85**/69 |
| **HF** | **1**/0 | **7**/3 | 0/**1** | **2**/1 | 9/9 | 3/3 | **5**/2 | **2**/1 |
| **MI+IHD** | 18/**19** | **7**/4 | **11**/10 | **3**/2 | 4/4 | **10**/9 | **2**/1 | **3**/1 |
| **DM** | **2**/1 | 0/0 | **5**/4 | **3**/2 | **4**/0 | **5**/1 | 7/**8** | 9/9 |
| **VA** | 0/**2** | 0/0 | 0/**1** | 0/0 | **1**/0 | **1**/0 | **1**/0 | **1**/0 |
| **CD** | 8/8 | 12/12 | 3/3 | **6**/2 | 0/**1** | 0/0 | 0/**1** | 0/**1** |
